# Automated machine learning‐based model for the prediction of delirium in patients after surgery for degenerative spinal disease

**DOI:** 10.1111/cns.14002

**Published:** 2022-10-18

**Authors:** Yu Zhang, Dong‐Hua Wan, Min Chen, Yun‐Li Li, Hui Ying, Ge‐Liang Yao, Zhi‐Li Liu, Guo‐Mei Zhang

**Affiliations:** ^1^ Outpatient Department The Second Affiliated Hospital of Nanchang University Nanchang China; ^2^ Medical Innovation Center the First Affiliated Hospital of Nanchang University Nanchang China; ^3^ Institute of Spine and Spinal Cord Nanchang University Nanchang China; ^4^ Department of Orthopedics The Second Affiliated Hospital of Nanchang University Nanchang China

**Keywords:** delirium, machine learning, model prediction, postoperative

## Abstract

**Objective:**

This study used machine learning algorithms to identify critical variables and predict postoperative delirium (POD) in patients with degenerative spinal disease.

**Methods:**

We included 663 patients who underwent surgery for degenerative spinal disease and received general anesthesia. The LASSO method was used to screen essential features associated with POD. Clinical characteristics, preoperative laboratory parameters, and intraoperative variables were reviewed and were used to construct nine machine learning models including a training set and validation set (80% of participants), and were then evaluated in the rest of the study sample (20% of participants). The area under the receiver‐operating characteristic curve (AUROC) and Brier scores were used to compare the prediction performances of different models. The eXtreme Gradient Boosting algorithms (XGBOOST) model was used to predict POD. The SHapley Additive exPlanations (SHAP) package was used to interpret the XGBOOST model. Data of 49 patients were prospectively collected for model validation.

**Results:**

The XGBOOST model outperformed the other classifier models in the training set (area under the curve [AUC]: 92.8%, 95% confidence interval [CI]: 90.7%–95.0%), validation set (AUC: 87.0%, 95% CI: 80.7%–93.3%). This model also achieved the lowest Brier Score. Twelve vital variables, including age, serum albumin, the admission‐to‐surgery time interval, C‐reactive protein level, hypertension, intraoperative blood loss, intraoperative minimum blood pressure, cardiovascular‐cerebrovascular disease, smoking, alcohol consumption, pulmonary disease, and admission‐intraoperative maximum blood pressure difference, were selected. The XGBOOST model performed well in the prospective cohort (accuracy: 85.71%).

**Conclusion:**

A machine learning model and a web predictor for delirium after surgery for the degenerative spinal disease were successfully developed to demonstrate the extent of POD risk during the perioperative period, which could guide appropriate preventive measures for high‐risk patients.

## Graphical abstract

A machine learning model and a web predictor for delirium after surgery for degenerative spinal disease are successfully developed to demonstrate the extent of POD risk during the perioperative period, which could guide appropriate preventive measures for high‐risk patients.

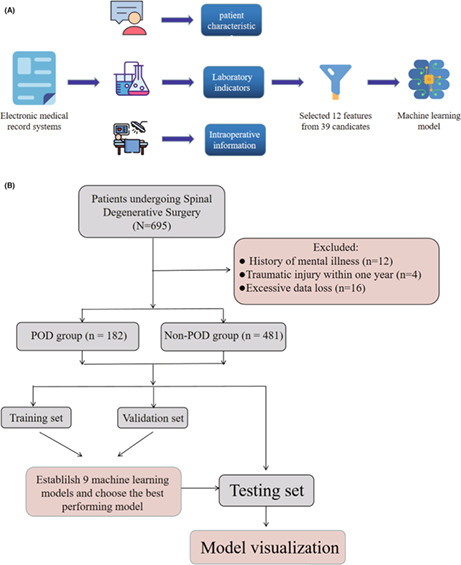

## 1 INTRODUCTION

Postoperative delirium (POD) is an acute state of confusion caused by reversible changes in the central nervous system caused by an underlying systemic disturbance. The occurrence usually occurs between a few hours and a few days after surgery, and mainly manifests as a significant loss of functional ability, a decline in consciousness, attention disorder, or thinking disorders.^1^ The total annual healthcare costs associated with delirium and its complications are estimated to be more than $164 billion.^2^ Since delirium is highly preventable,^3^, ^4^ interventions are increasingly targeted to reduce its complications and costs.

From the nineties of the last century up to today, spinal surgery continues to increase,^5^ but the occurrence of postspinal delirium is not uncommon.^6^ There is a paucity of studies on POD among patients undergoing surgery for degenerative spinal lesions,^7^, ^8^ and early models of disease progression in these patients used a single statistical method with limited predictive power.^9^, ^10^, ^11^ Using machine learning techniques to establish disease prediction models can enhance the predictive power of these models, as has been reported.^12^, ^13^

Machine learning technology was used in the present study to extract the preoperative and intraoperative clinical data of 663 patients who underwent surgery under general anesthesia, and nine different predictive models for POD were developed. Finally, we compared these models and selected the optimal predictive model capable of assisting in detecting and diagnosing patients at high risk of POD. Additionally, to make the selected model more available, a website calculator was established to help clinicians in their daily application.

## 2 METHODS

### 2.1 Data source and extraction

This study was designed as a retrospective study, which was performed in compliance with the STROBE Guidelines.^14^ We recruited patients who underwent surgery for degenerative spinal pathologies, including cervical disk herniation, cervical spinal stenosis, lumbar disk herniation, and lumbar spinal stenosis, at the First Affiliated Hospital of Nanchang University from January 2018 to October 2021. This study was approved by the Ethics Committee of the First Affiliated Hospital of Nanchang University under the accreditation number (2022) YYL‐K (4–013). All patient data were anonymized throughout the study. No identifiable data of patients were recorded. Given that this study was purely observational, written consent was not required. We included patients that underwent surgery at our hospital as indicated by the inefficacy of conservative treatment for degenerative spinal conditions. Patients with a history of severe psychiatric disease, preoperative delirium, severe traumatic brain injury, or severe organ damage within the last 12 months were excluded from the study.

The accuracy of the model was further validated by prospectively collecting data from patients undergoing surgery for degenerative spine disease from December 2021 to February 2022 at the First Affiliated Hospital of Nanchang University.

### 2.2 Delirium assessment

Delirium was assessed using rigorous techniques. In this trial, the diagnosis of delirium was made by a multidisciplinary consensus panel based in accordance with the Diagnostic and Statistical Manual of Mental Disorders (Fourth Edition) criteria using several data sources, including the confusion assessment method,^15^ the Delirium Rating Scale‐Revised‐98 (DRS),^16^ digit span, a review of medical records, and family/nursing staff interviews. We judged this by the occurrence of delirium in the first 5 days after surgery.^17^ The evaluation of delirium was carried out by a qualified psychiatrist at the First Affiliated Hospital of Nanchang University who was blinded to both the patient's perioperative characteristics and the process of data entry and statistical analysis.

### 2.3 Model input features

We selected thirty‐nine potential features, including basic patient characteristics: age, sex, weight, education, hypertension, diabetes, history of pulmonary disease (PD), history of cardiovascular‐cerebrovascular disease (CCD), visual impairment, hearing impairment, history of alcoholism, smoking history, admission‐to‐surgery time interval (ASTI), blood pressure on admission, history of previous surgery; preoperative laboratory data including blood group, white blood cell count, red blood cell count, red blood cell ratio, hemoglobin level, serum C‐reactive protein (CRP), erythrocyte sedimentation rate (ESR), serum albumin, AST titer, ALT titer, serum creatinine, blood urea nitrogen, serum potassium, serum sodium, serum chloride, and serum calcium levels; procedure‐specific information such as the American Society of Anesthesiologists (ASA) classification, volume of blood transfused, intraoperative minimum blood pressure (IMBP), admission‐intraoperative maximum blood pressure difference (AIMBPD), number of operative segments, intraoperative blood loss (IBL), duration of surgery, and intraoperative cerebrospinal fluid leakage. Then, POD features were selected using the least absolute shrinkage and selection operator (LASSO).^18^

To obtain the best predictive performance, nine models, including the eXtreme Gradient Boosting (XGBOOST) algorithm, Logistic regression (LR), RandomFofest (RF), AdaBoost, GaussianNB (GNB), ComplementNB (CNB), Multi‐layer Perceptron (MLP), Support Vector Machine (SVM), and K‐Nearest Neighbor machine (KNN) learning models, were built.

### 2.4 Sample size and statistical analysis

For the two‐class prediction model, the sample size calculation was obtained as described in a previous study,^19^ as follows: $N=\exp\left(\frac{-0.508+0.259\ln\left(\varphi \right)+0.504\ln\left(P\right)-\ln\left(\text{MAPE}\right)}{0.544}\right)$. According to the above formula, the minimum sample size is estimated to be 480. To meet these requirements, we randomly split the study population (*n* = 663) into the training set, validation set, and testing set.

All analyses were performed using Python language. The normality of the distribution of continuous variables was tested using the Shapiro–Wilk test. Normally distributed continuous variables were expressed as the mean ± standard deviation (SD) and compared using the independent‐sample *t*‐test. Skewed continuous variables were presented as the median and interquartile range (IQR) and compared using the Mann–Whitney U test. Categorical variables were presented as frequencies and percentages and analyzed using either the chi‐square test or Fisher's exact probability test. At last, the most important features were filtered via LASSO regression analysis, and nine models were developed based on their set of features.

The selection of model hyperparameters used ten‐fold cross‐validation on training datasets. Cross‐validation guaranteed a better assessment of model performance by averaging metrics over multiple trials. The application of missing data imputation is described as follows: If the percentage of missing values was >20%, it was excluded from the final completed dataset, and if this percentage was <20%, the random forest regression method was used for imputation.

Discrimination and calibration were used to verify the predictive ability of the model. Clinical decision curve analysis (DCA)^20^ evaluated the clinical utility of the model. The AUROC and Brier score were the measurements of discrimination. After the best model was selected, the Shapley Additive exPlanations (SHAP) package^21^ in Python was used to show the relationship between the importance of each feature. Finally, the best model was applied to visualize prospective validations.

## 3 RESULTS

### 3.1 Patient characteristics

**FIGURE 1:**
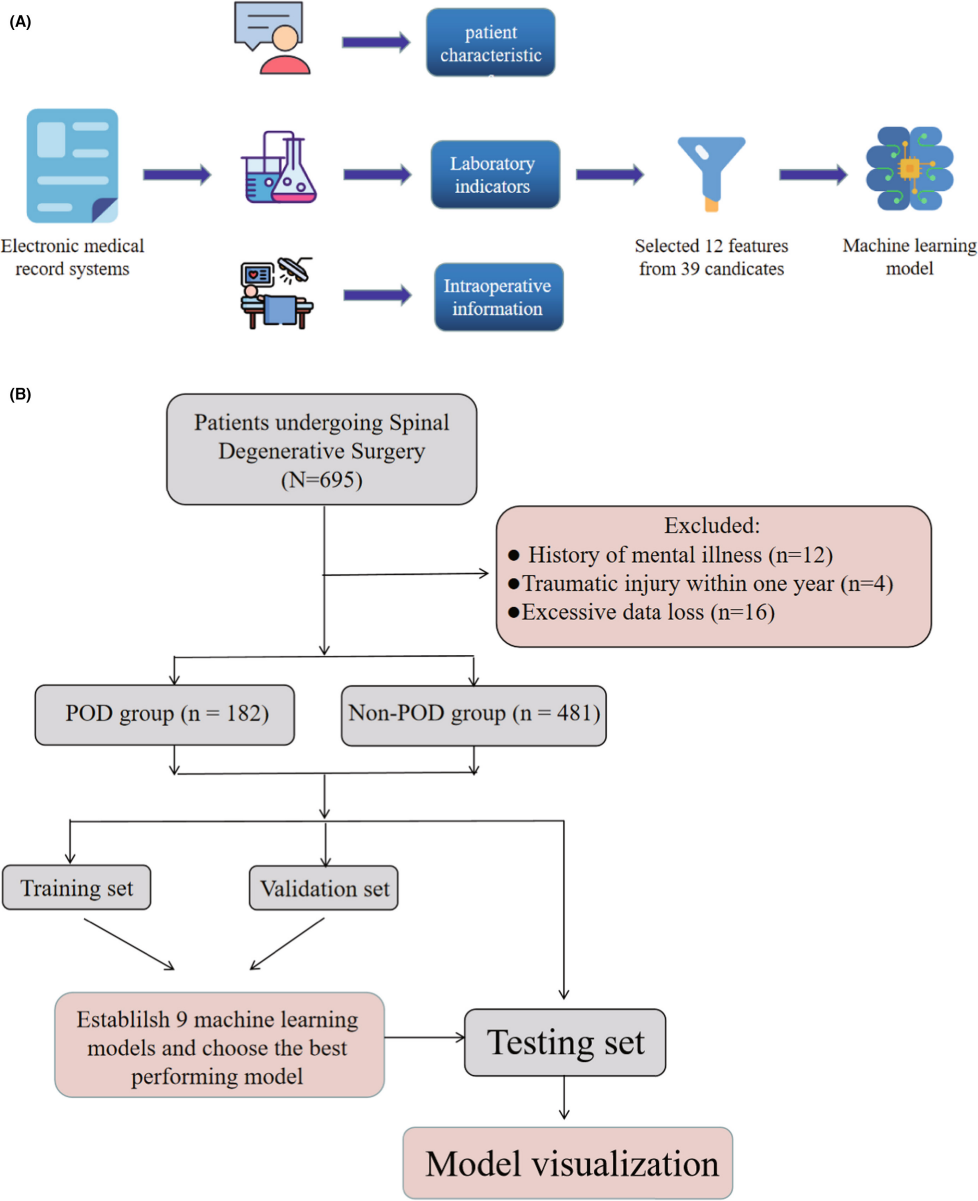
Model‐making process and flowchart of the study procedure. (A) This figure shows how the data were obtained from electronic medical record systems, and the collection of data on all study variables, including demographic characteristics, laboratory indicators, and intraoperative information. Data on a total of 39 preoperative variables were collected, 12 of which were selected. The 12 variables were used to establish the machine learning models. (B) Flowchart of our study procedure

A total of 663 patients were included in this study. Detailed information on the demographic characteristics of the study participants who underwent surgery for degenerative spinal pathologies are shown in Table 1. The comparison of preoperative and intraoperative variables between POD group and non‐POD group can be found in Table 2. Among those screened, the rate of POD was approximately 27.45%. The study variables, including preoperative CRP and preoperative serum albumin, had few missing values. The study population (*n* = 663) was randomly divided into training set and validation set, which were used to establish the predictive models. and testing set, which was used to further validate the predictive models. Our study participants were divided into the POD group (*n* = 182) and non‐POD group (*n* = 481) according to whether or not they experienced delirium within the first 5 days after surgery.^17^ The flowchart for patient recruitment is shown in Figure 1. There was no statistically significant difference between the patient characteristics in the training and testing datasets.

**TABLE 1:** Demographic characteristics of the study participants who underwent surgery for degenerative spinal pathologies

Variables	All (*N* = 663)	POD group (*n* = 182)	Non‐POD group (*n* = 481)	*p*‐Value
Age, median (Q1, Q3)	58 (49, 67)	68 (63, 75)	54 (46, 63)	<0.001
Sex, *n*%				
Female	274 (41.327)	69 (37.912)	205 (42.620)	0.272
Male	389 (58.673)	113 (62.088)	276 (57380)	
Weight, median (Q1, Q3)	60 (54, 69)	60 (53, 65)	60 (54, 70)	0.043
Education degree				
Illiteracy	22 (3.318)	13 (7.143)	9 (1.871)	0.008
Junior high school education and below	503 (75.867)	137 (75.275)	366 (76.091)	
High school education	81 (12.217)	16 (8.791)	65 (13.514)	
University degree and above	57 (8.597)	16 (8.791)	41 (8.524)	
Blood group, *n*%				
A	221 (33.333)	58 (31.868)	163 (33.888)	0.179
B	175 (26.395)	59 (32.418)	116 (24.116)	
O	217 (32.730)	53 (29.121)	164 (34.096)	
AB	50 (7.541)	12 (6.593)	38 (7.900)	

Abbreviations: SD, standard deviation.

**TABLE 2:** Comparison of preoperative and intraoperative variables between POD group and non‐POD group

Variables	All (*N* = 663)	POD group (*n* = 182)	Non‐POD group (*n* = 481)	*p*‐Value
Hypertension, *n*%	260 (39.216)	126 (69.231)	134 (27.859)	<0.001
Diabetes, *n*%	54 (8.145)	22 (12.088)	32 (6.653)	0.022
Pulmonary disease, *n*%	27 (4.072)	18 (9.890)	9 (1.871)	<0.001
CCD, *n*%	53 (7.994)	37 (20.330)	16 (3.326)	<0.001
Visual impairment, *n*%	1 (0.151)	0 (0.000)	1 (0.208)	<0.001
Hearing impairment, *n*%	2 (0.302)	2 (1.099)	0 (0.000)	<0.001
Alcohol, *n*%	25 (3.771)	18 (9.890)	7 (1.455)	<0.001
Smoking, *n*%	51 (7.692)	32 (17.582)	19 (3.950)	<0.001
ASTI, median (Q1, Q3)	4 (3.000, 5.000)	5 (3.000, 7.000)	3 (2.000, 5.000)	<0.001
Blood pressure on admission, median (Q1, Q3)	130 (117, 141)	135 (125, 147)	127 (115, 139)	<0.001
History of previous surgery, *n*%	181 (27.300)	67 (36.813)	114 (23.701)	<0.001
WBC, median (Q1, Q3)	6.080 (4.990, 7.300)	6.120 (4.930, 7.220)	6.030 (4.990, 7.330)	0.831
RBC, mean (SD)	4.392 (0.541)	4.202 (0.536)	4.464 (0.526)	<0.001
RBC ratio, median (Q1, Q3)	0.408 (0.378, 0.441)	0.393 (0.366, 0.423)	0.414 (0.383, 0.448)	<0.001
Hemoglobin, median (Q1, Q3)	133 (123, 145)	128 (119, 140)	135 (125, 148)	<0.001
CRP, median (Q1, Q3)	1.480 (0.580, 3.140)	2.600 (1.200, 5.320)	1.270 (0.480, 2.520)	<0.001
ESR, median (Q1, Q3)	7 (4， 12)	9 (4， 18)	6 (4. 11)	<0.001
Albumin, median (Q1, Q3)	40.400 (38.100， 42.700)	38.000 (35.300, 40.100)	41.300 (39.100， 43.500)	<0.001
AST, median (Q1, Q3)	18 (13,28)	17 (12, 25)	18 (13, 29)	0.041
ALT, median (Q1, Q3)	20.000 (16.800, 25.000)	20.000 (17.000, 24.000)	20 (16.000, 26.000)	0.829
Cr, median (Q1, Q3)	66.700 (56.500, 78.100)	70.000 (61.200, 84.500)	65.900 (55.500, 76.600)	<0.001
BUN, median (Q1, Q3)	5.640 (4.720, 6.960)	6.180 (5.040, 7.650)	5.510 (4.600, 6.710)	<0.001
K+, median (Q1, Q3)	3.900 (3.690, 4.130)	3.870 (3.620, 4.150)	3.910 (3.710, 4.130)	0.220
Na+, median (Q1, Q3)	140.700 (139.300, 142.100)	141.000 (139.800, 142.900)	140.600 (139.300, 141.900)	0.004
Cl‐, median (Q1, Q3)	104.900 (103.300, 106.640)	105.200 (102.900, 107.000)	104.830 (103.390, 106.480)	0.304
Ca+, median (Q1, Q3)	2.270 (2.200, 2.350)	2.260 (2.180, 2.340)	2.280 (2.210, 2.350)	0.196
ASA degree, *n*%				
II	158 (23.831)	17 (9.341)	141 (29.314)	<0.001
III	501 (75.566)	163 (89.560)	338 (70.270)	
IV	4 (0.603)	2 (1.099)	2 (0.416)	
Volume of blood transfusion, mean (SD)	43.881 (216.847)	102.775 (371.026)	21.550 (104.125)	0.004
IMBP, median (Q1, Q3)	100 (90, 105)	95 (85, 100)	100 (90, 105)	<0.001
AIMBPD, median (Q1, Q3)	32 (19, 45)	40 (29, 53)	28 (16, 40)	<0.001
Operative segments, *n*%				
1	342 (51.584)	60 (32.967)	282 (58.628)	<0.001
2	199 (30.015	64 (35.165)	135 (28.067)	
3	100 (15.083)	43 (23.626)	57 (11.850)	
4 and above	22 (3.318)	15 (8.242)	7 (1.455)	
IBL, median (Q1, Q3)	200 (100, 350)	300 (200, 400)	200 (100, 300)	<0.001
Duration of surgery, *n*%	160 (120, 205)	175 (135, 225)	150 (110, 200)	<0.001
Cerebrospinal fluid leak, *n*%	17 (2.564)	7 (3.846)	10 (2.079)	0.199

Abbreviations: AIMBPD, admission‐intraoperative maximum blood pressure difference; ALT, alanine aminotransferase; AST, aspartate aminotransferase; ASTI, admission‐to‐surgery time interval; BUN, blood urea nitrogen; CCD, cardiovascular‐cerebrovascular disease; Cr, creatinine; CRP, C‐reactive protein; IBL, intraoperative blood loss; IMBP, intraoperative minimum blood pressure; RBC, red blood cell; SD, standard deviation; WBC, white blood cell.

### 3.2 Key variables

**FIGURE 2:**
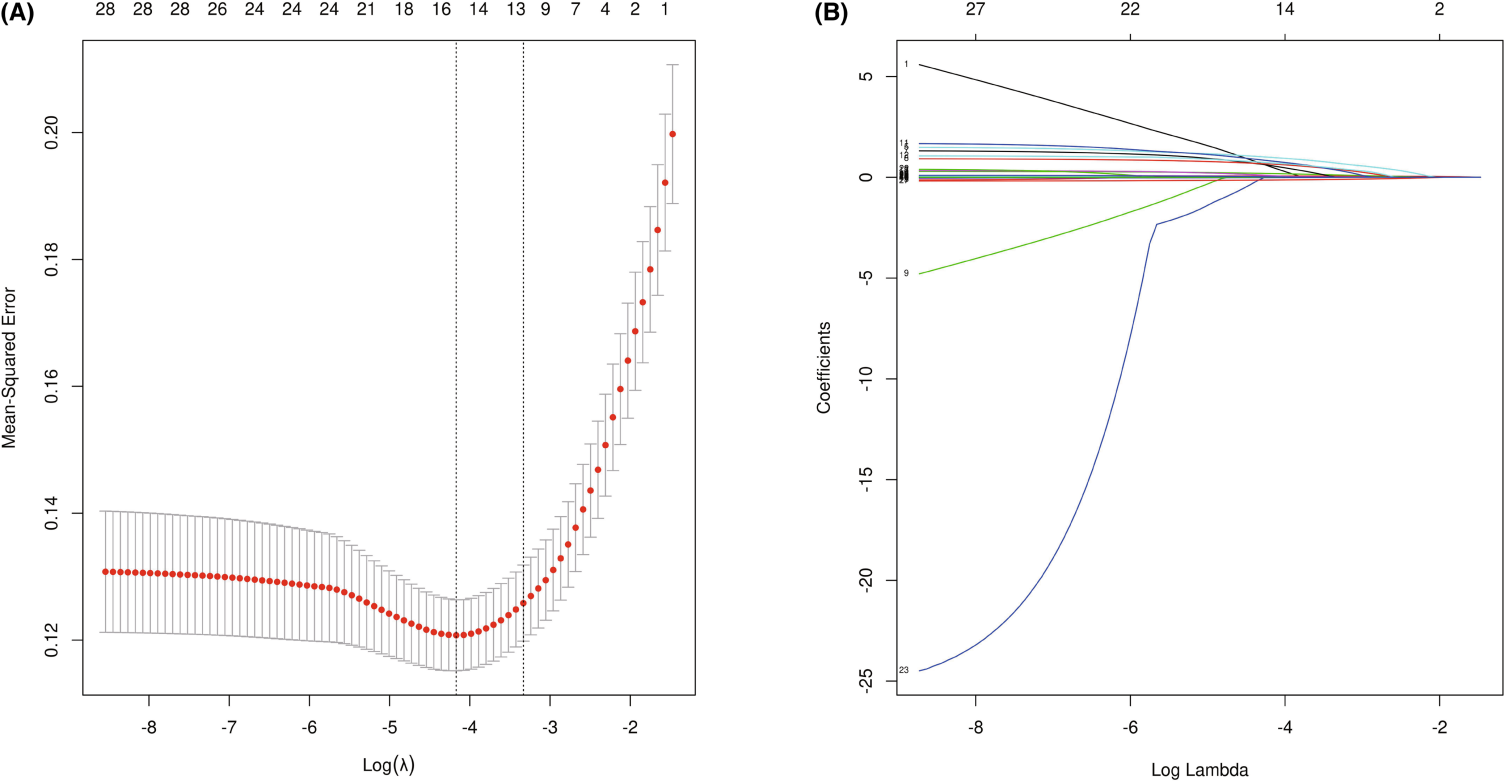
A, B Demographic and clinical feature selection using the LASSO regression

In the LASSO model, a vertical line was drawn at the value selected using the ten‐fold cross‐validation, where a suitable lambda resulted in 12 features with nonzero coefficients (Figure 2A,B). As expected, patients with advanced age, hypertension, history of smoking, CCD, PD, and history of alcoholism, longer ASTI, lower preoperative albumin, and minimum intraoperative blood pressure, greater intraoperative blood loss, higher preoperative CRP, and larger AIMBPD were more likely to experience POD.

### 3.3 Model performance

**FIGURE 3:**
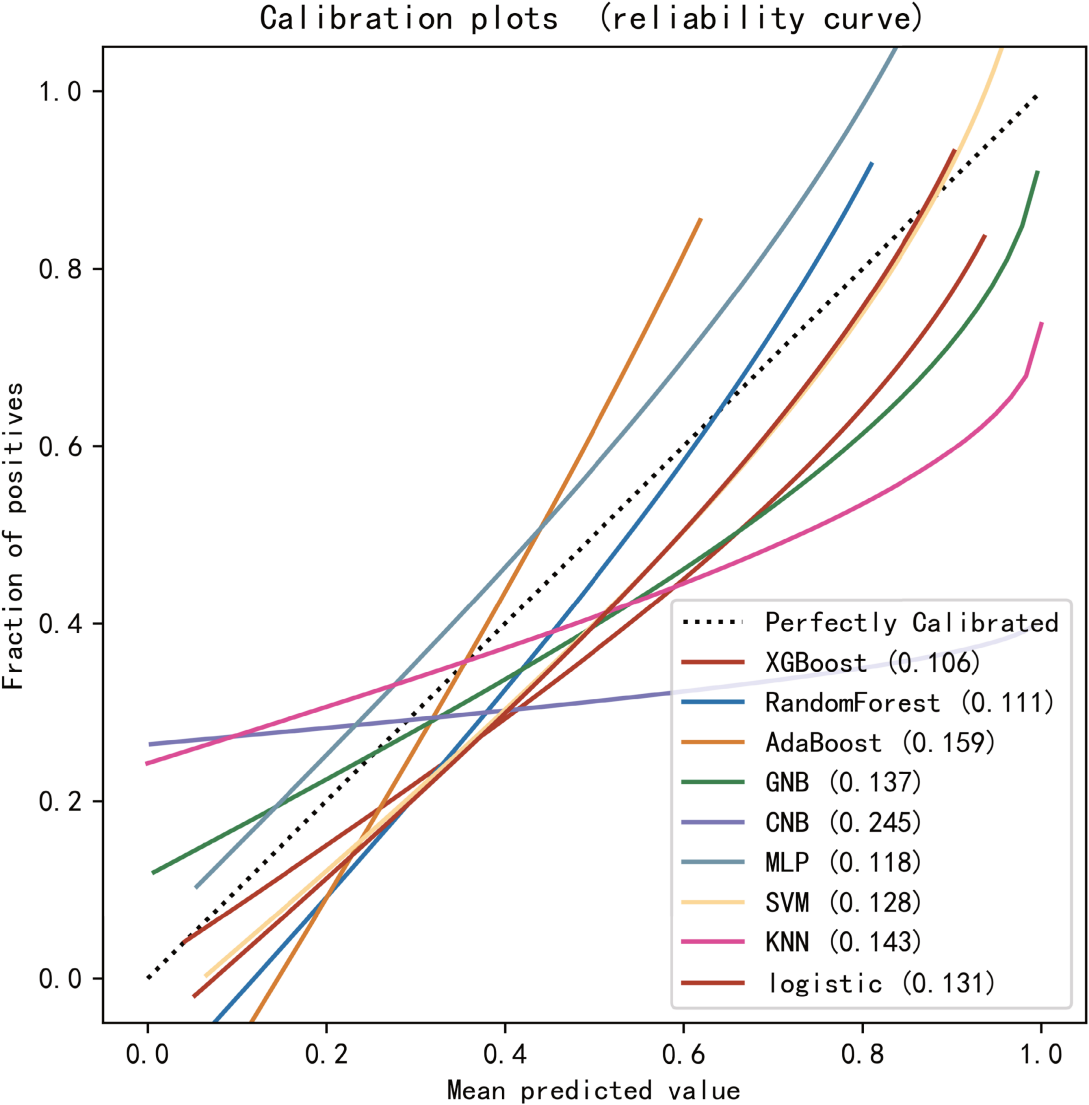
Calibration plots of nine models. The XGBoost achieved lower (better) Brier scores compared with the other models.

**FIGURE 4:**
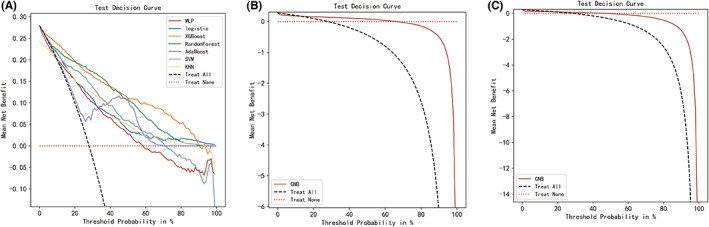
Decision curve analysis for nine machine learning models. The XGBoost model can serve as the best diagnostic tool for postoperative delirium.

After identifying these 12 variables, machine learning models were used to predict POD after surgery. AUROC, Brier Scores, and DCA are important indicators used to evaluate prediction models. The XGBoost achieved a much lower and superior Brier score compared with the other models. The calibration plots of the nine models are shown in Figure 3. DCA indicated that the XGBoost model could serve as the best diagnostic tool for POD (Figure 4).

**FIGURE 5:**
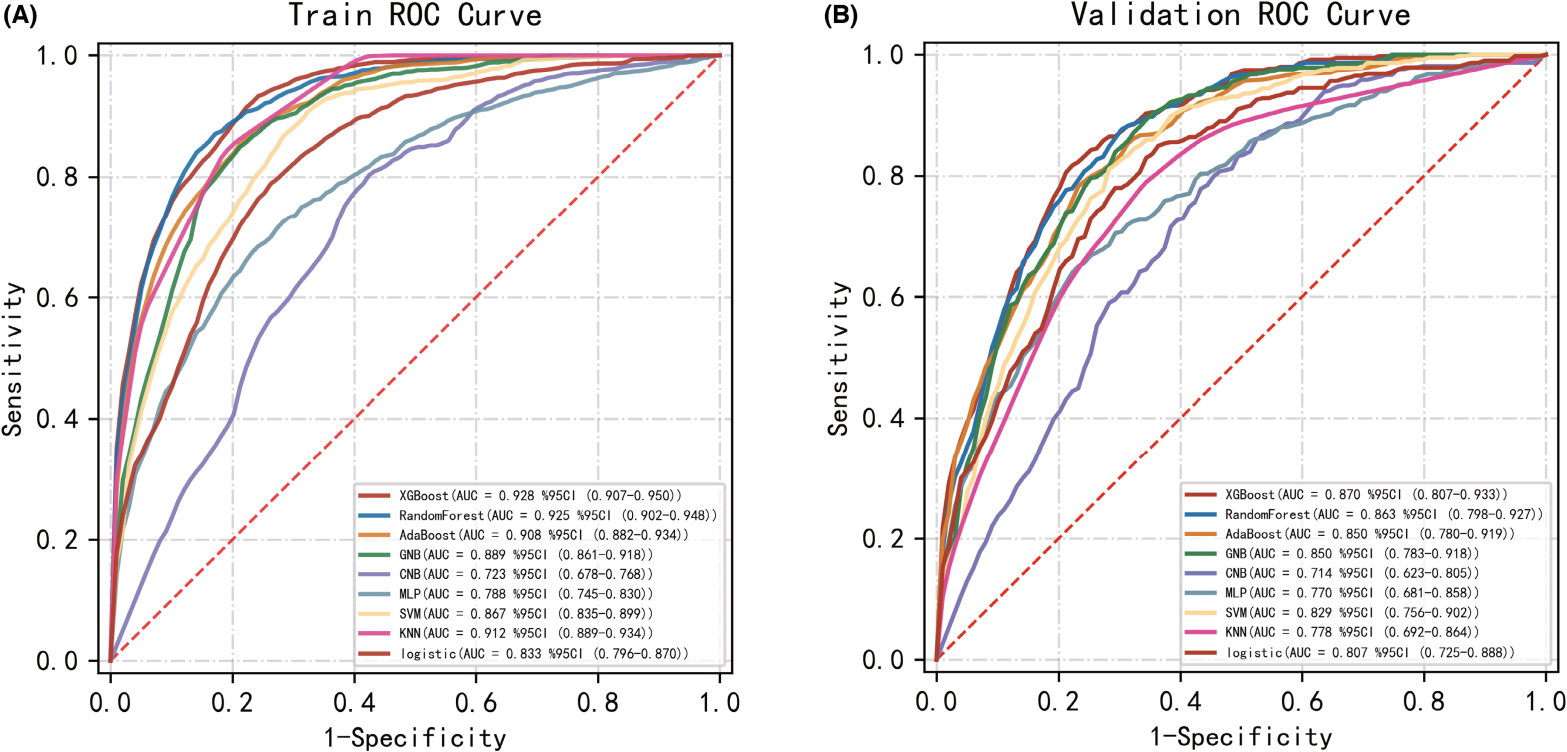
Receiver‐operating characteristic curves for nine machine learning models. The XGBoost model achieved a larger (better) AUROC compared with the other models.

**FIGURE 6:**
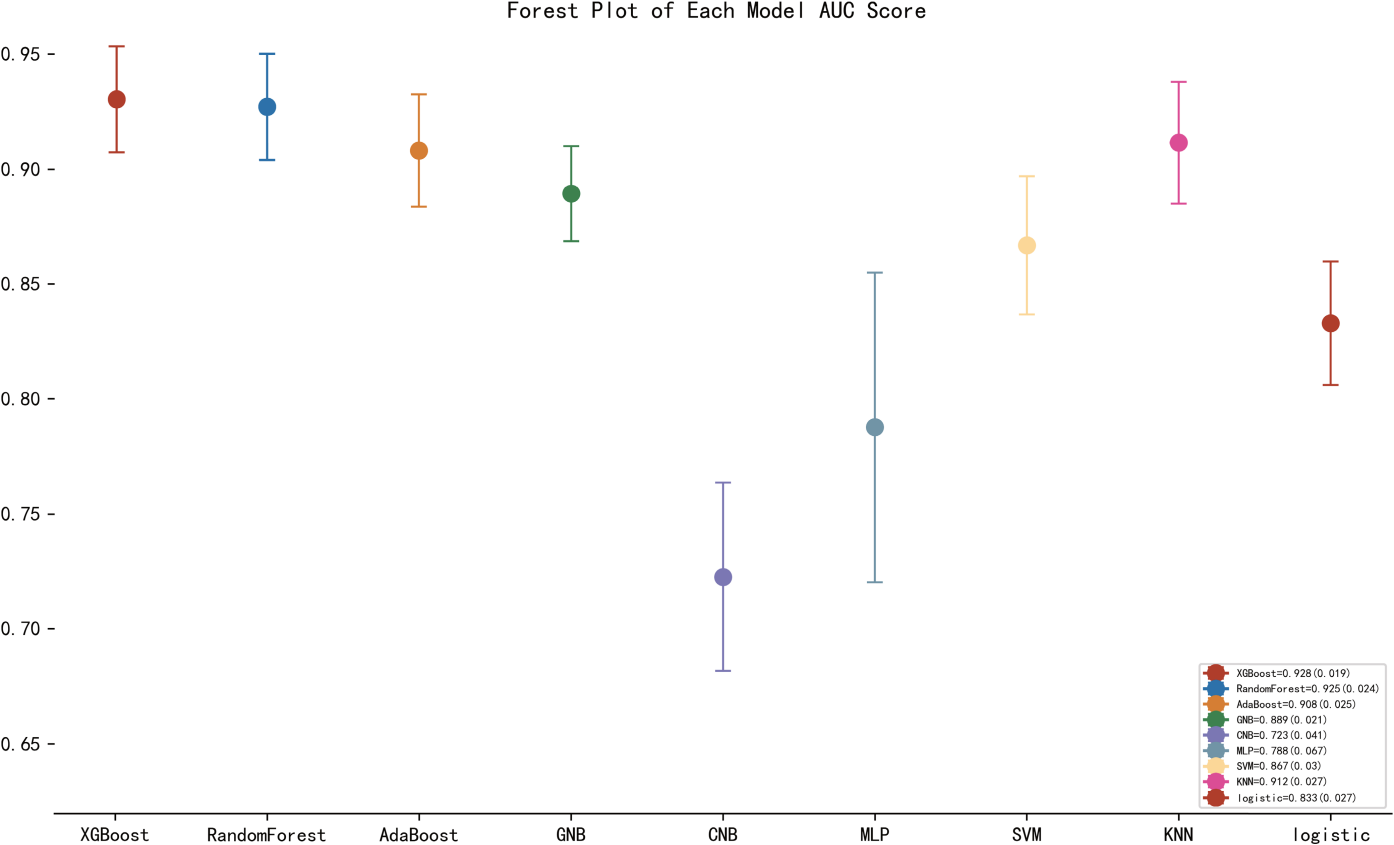
Forest plot of the AUC Score of the nine models. The XGBoost model achieved a smaller (better) standard deviation (SD) compared with the other models.

The XGBoost model also achieved a larger (better) AUROC compared with the other models (Figure 5). Based on the AUROC of the nine models, we made a forest plot of the AUC score of the multiple models. Nine models were seen after using 10 cross‐validations: the standard deviation SD of the AUC score of the XGBoost model is 0.019, which is smaller than the other eight models, indicating that the XGBoost model has the most stable performance. Based on the above aspects, we can conclude that the XGBoost model significantly outperformed eight other machine learning models (Figure 6). The values in the training datasets are found in Table 3. The values in the validation set are found in Table 4. According to the Youden Index, which is defined as sensitivity + specificity − 1, the best cut‐off of prediction probabilities of the XGBoost model was 29.53%. For the testing dataset, the data of 132 patients were collected to validate the performance of the established XGBoost model. The following values in the testing group were found: XGBoost model (AUC = 87.9%, 95% CI: 81.9%–94.0%), accuracy = 0.812, sensitivity = 0.909, specificity = 0.770, F1 scores = 0.713.

**TABLE 3:** Performance metrics for nine models in the training dataset

Model	AUC (95%) CI	Accuracy	Sensitivity	Specificity	Youden Index	F1 score
XGBoost	0.928 (0.907–0.950)	0.828	0.904	0.803	0.707	0.745
Logistic	0.833 (0.796–0.870)	0.743	0.827	0.716	0.543	0.64
RandomForest	0.925 (0.902–0.948)	0.827	0.872	0.84	0.712	0.761
AdaBoost	0.908 (0.882–0.934)	0.819	0.851	0.808	0.659	0.723
GNB	0.889 (0.861–0.918)	0.806	0.862	0.788	0.65	0.711
CNB	0.723 (0.678–0.768)	0.645	0.8	0.588	0.388	0.553
MLP	0.788 (0.745–0.830)	0.746	0.711	0.76	0.471	0.602
SVM	0.867 (0.835–0.899)	0.753	0.907	0.697	0.604	0.67
KNN	0.912 (0.889–0.934)	0.824	0.845	0.813	0.658	0.768

Abbreviations: CNB, ComplementNB; GNB, GaussianNB; KNN, K‐nearest neighbor; MLP, multilayer perceptron; SVM, support vector machine; XGBOOST, eXtreme Gradient Boosting.

**TABLE 4:** Performance metrics for nine models in the validation dataset

Model	AUC (95%) CI	Accuracy	Sensitivity	Specificity	Youden Index	F1 score
XGBoost	0.870 (0.807–0.933)	0.774	0.861	0.773	0.634	0.673
Logistic	0.807 (0.725–0.888)	0.708	0.805	0.725	0.53	0.601
RandomForest	0.863 (0.798–0.927)	0.772	0.819	0.793	0.612	0.666
AdaBoost	0.850 (0.780–0.919)	0.763	0.821	0.754	0.575	0.656
GNB	0.850 (0.783–0.918)	0.753	0.870	0.731	0.601	0.656
CNB	0.714 (0.623–0.805)	0.615	0.787	0.596	0.383	0.518
MLP	0.770 (0.681–0.858)	0.704	0.712	0.746	0.458	0.561
SVM	0.829 (0.756–0.902)	0.708	0.904	0.660	0.564	0.624
KNN	0.778 (0.692–0.864)	0.747	0.762	0.711	0.473	0.641

Abbreviations: CNB, ComplementNB; GNB, GaussianNB; KNN, K‐nearest neighbor; MLP, multilayer perceptron; SVM, support vector machine; XGBOOST, eXtreme Gradient Boosting.

**FIGURE 7:**
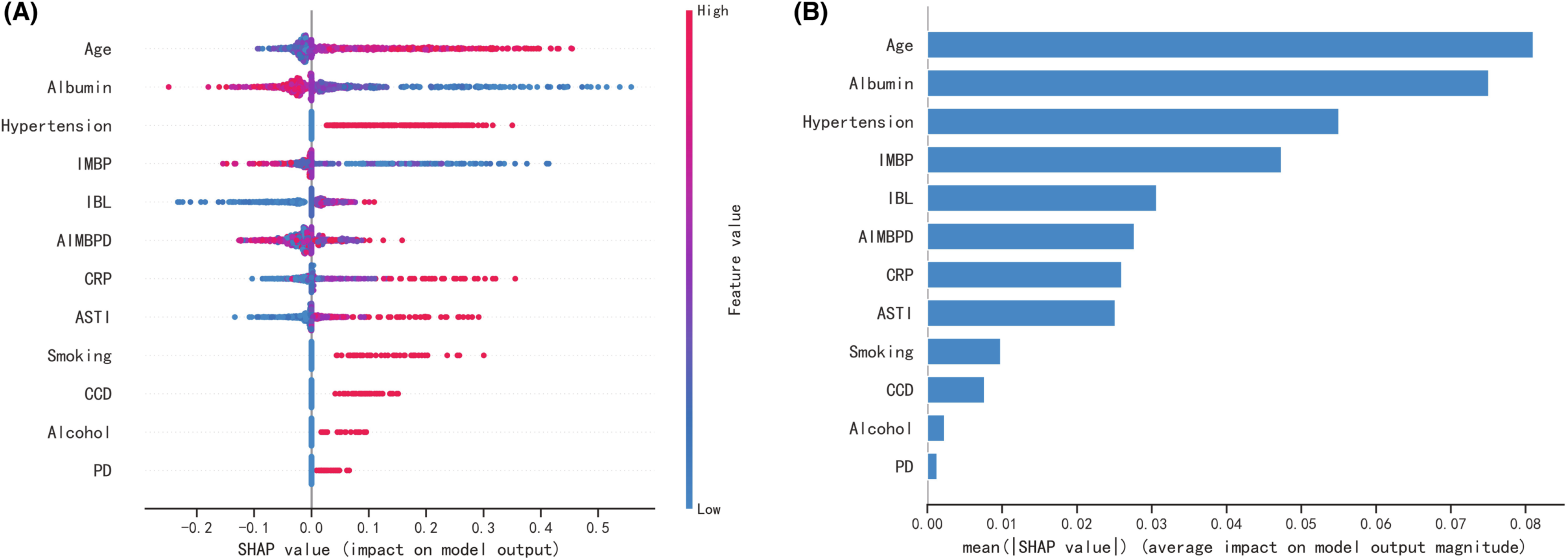
SHAP analysis of the XGBoost model. A visual representation of each feature of the XGBoost model, showing the relationship between the importance of each feature. The color represents the value of the variable, with red representing the larger value and blue representing the smaller value.

The SHAP package was used to analyze XGBoost model, showing the reflect the influence of each feature in the sample and also showing the positive and negative influences. The bar chart shows the relationship between the magnitude of the feature value and the predicted impact (Figure 7). Meanwhile, we did a sensitivity analysis using SALib, which is an open‐source library for sensitivity analysis based on python, and we found that the results were similar to those presented by the SHAP value (Table 5). The two‐class prediction outcome was generated based on the optimal cut‐off value of the optimal model. For the application of the XGBoost model, the best cut‐off of the prediction probability of the proposed model was 29.53%. If the model predicted a probability > 29.53%, then the patients who underwent surgery had a higher risk of developing POD. At this moment, nursing staff and doctors should pay closer attention to patients with these characteristics.

**TABLE 5:** Sensitivity analysis of XGBoost model

Features	Sensitivity Index	95% CM
Age	0.597310	0.015986
Albumin	0.011097	0.001418
Hypertension	0.060957	0.002611
IMBP	0.131188	0.004410
IBL	0.233050	0.007096
AIMBPD	0.003855	0.000158
CRP	0.018204	0.001480
ASTI	0.013549	0.000701
Smoking	0.001563	0.000076
CCD	0.001141	0.000231
Alcohol	0.000285	0.000176
PD	0.000096	0.000137

Abbreviations: AIMBPD, admission‐intraoperative maximum blood pressure difference; ASTI, admission‐to‐surgery time interval; CCD, cardiovascular‐cerebrovascular disease; CM, confidence measure; IBL, intraoperative blood loss; IMBP, intraoperative minimum blood pressure; PD, pulmonary disease.

### 3.4 Application of the model

**FIGURE 8:**
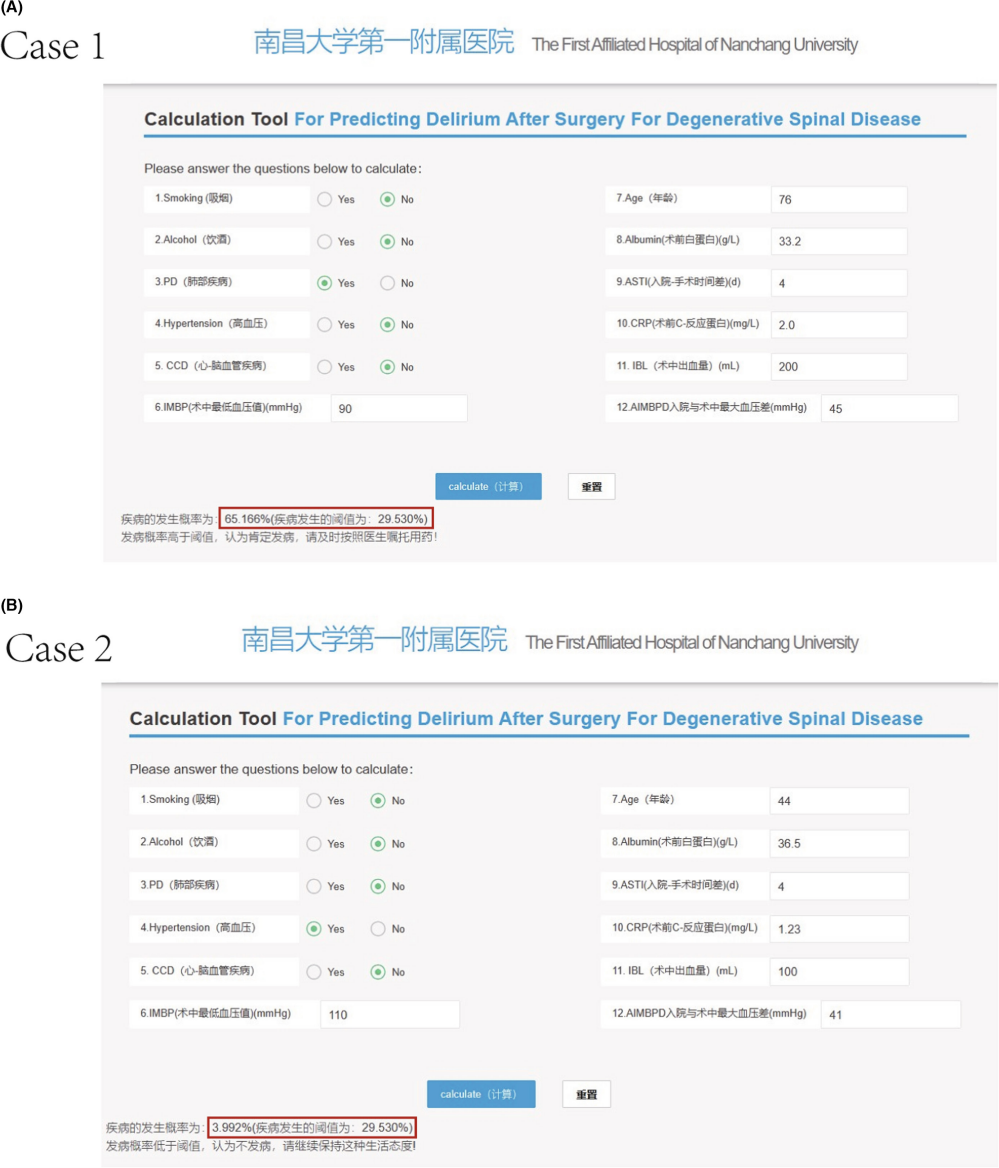
Cases of website usage. Entering the input value determined the POD requirements and displayed how each value contributed to the prediction. (A) Case 1 POD will occur; (B) Case 2 POD will not occur.

A typical patient's preoperative and intraoperative information was inputted into the model, for example, age: 76 years, albumin: 33.2 g/L, ASTI: 4 days, CRP: 2.00 mg/L, IBL: 200 ml, IMBP: 115 mmHg, AIMBPD: 45 mmHg, smoking: No, alcohol: No, PD: Yes, hypertension: No, CCD: No. The model predicted that the risk of POD in this patient was 65.166%, indicating that the patient was at high risk of POD; indicating that medical staff should prepare for treatment and care in advance (Figure 8A). Using the preoperative and intraoperative information of another patient in the model: age: 44 years, albumin: 36.5 g/L, ASTI: 4 days, CRP: 1.23 mg/L, IBL: 100 ml, IMBP: 110 mmHg, AIMBPD: 41 mmHg, smoking: No, alcohol: No, PD: No, hypertension: Yes, CCD: No, the predicted probability of POD in this patient was 3.992%, indicating that the patient was at low risk of developing POD (Figure 8B). Furthermore, a web‐based tool was established for clinicians to use the proposed model (available at: http://121.89.246.238/model/prediction/1).

### 3.5 Prospective validation

The data of 49 patients were prospectively collected for validation, among which 18.37% (9/49) experienced POD. The accuracy of the proposed model on the prospective dataset was 85.71%. The sensitivity and specificity for the prospective validation sample were 0.778 and 0.875, respectively. For two patients that experienced POD, the model predicted a negative outcome. The model predicted five patients who did not experience POD as positive, and the others had probabilities of developing POD (3.18%–20.50%) that were lower than the cut‐off value.

## 4 DISCUSSION

Few models are available for predicting delirium after surgery for patients with spinal degenerative disease.^22^, ^23^, ^24^ This study presents a novel approach using machine learning algorithms to predict delirium in these patients. The XGBoost machine learning model accurately predicted POD and showed greater discrimination and satisfactory specificity and sensitivity than the other models developed in this study.

Postoperative delirium occurs in 11%–51% of patients after surgery.^25^ The incidence of POD in this study was 27.45%. The occurrence of POD is caused by a variety of factors, and the probability of its occurrence may also be related to the type of surgery and perioperative intervention.^26^ Therefore, for patients with different diseases, there are certain differences in the occurrence probability of POD. In patients, around 30%–40% of delirium cases are thought to be attributable to modifiable risk factors and are therefore preventable.^27^ Prophylactic low‐dose infusion of dexmedetomidine significantly reduced the incidence of delirium in elderly patients during the first 7 days of admission to the ICU after noncardiac surgery.^28^ In deep anesthesia，the neurometabolic profile of isoflurane appears to be superior to that of propofol, which has been shown to impair the mitochondrial respiratory chain，isoflurane may therefore reduce neurological complications.^29^ Jie Zhang et al^30^ found that the composition of the gut microbiota was different between POD and non‐POD mice and concluded that targets gut microbiota could provide a novel alternative for POD treatment.

Researchers have conducted a number of clinical studies to identify biomarkers accurately predicting PODs, such as the evaluation of plasma tau,^31^ the S‐100β protein,^32^ amyloid,^33^ adiponectin^34^ levels, and the level of PGRN in the cerebrospinal fluid.^35^ Researchers are also trying to determine how to reduce the prevalence of the condition. Although POD can be predicted relatively well by these biomarkers, due to the complex sampling methods required and high associated costs, they are difficult to use in the clinical setting. Therefore, disease prediction models may provide a solution for the identification of high‐risk patients and the prevention of POD allowing clinicians to take measures to reduce the probability of its occurrence. Thus, the management of patients would be improved, and consequently, this would improve patient outcomes and reduce morbidity and costs. Thus, it is of great importance to predict POD and take appropriate measures immediately after surgery.^36^

There are many approaches to the construction of POD prediction models; however, many mathematical terms are always involved^37^ and are transformed into mathematical formulas, which limit the availability of prediction models. The model proposed in this study may help clinicians identify patients by predicting those at high risk of developing POD. To further enhance the simplicity of the model, this study also produced an online tool that will greatly improve the efficiency of the application of the model. It is expected that for each case of POD that is avoided, both the patient and financial benefits achieved may be significant given the large number of patients undergoing spinal surgery.

Additionally, two examples were given to illustrate how the model was able to predict POD and evaluate the relative importance of each variable for the clinician. With millions of spinal surgeries taking place each year, the findings could help give surgeons information about respective probabilities to develop POD of patients after surgery.

Previous studies have reported that postoperative IL‐6^32^ and IL‐8^31^ were associated with an increased risk of POD. However, preoperative and intraoperative information should be used to predict POD to define risk; to avoid the occurrence of POD on the night of surgery when it is too late to take action based on postoperative findings.

In this study, we established a predictive model and incorporated the following 12 variables into its construction: age, preoperative serum albumin, ASTI, preoperative CRP level, hypertension, IBL, IMBP, CCD, history of smoking, history of alcohol consumption, PD, and the AIMBPD. The optimal predictive model was made available as a Web‐based online tool. The use of online calculation models to estimate the risk of POD is a new concept finding greater application among clinicians. The XGBoost model performed well, with AUCs of 92.8% and 87.9% in the training and testing datasets, respectively. The calibration of the XGBoost model showed good agreement between the predicted outcome and the actual observed outcome by using Brier scores. For the application of this model, the best cut‐off of prediction probabilities of the proposed model was 29.53%. If this value was exceeded, patients undergoing surgery were at a higher risk of developing POD. This predictive model can be used as a tool to screen patients for POD. Therefore, targeted interventions can be carried out in advance for high‐risk patients.

A total of 12 variables were included in the XGBoost model analysis. Studies assessing the risk of POD have also demonstrated the vital role of age and some basic systemic diseases in predicting POD.^1^, ^38^ Our findings were consistent with those of previous studies. Low preoperative serum albumin may increase the incidence of POD in patients, probably because albumin reflects their nutritional status, and compared with patients having high nutritional status, these patients have difficulty in tolerating the shock of surgery and are more prone to POD. IBL and intraoperative hypotension were also associated with the development of POD, as confirmed by this study. Furthermore, this study also found other variables that increased the risk of POD, such as the time interval between ASTI, with a cut‐off value of 5.5 days. The participants of this study were patients scheduled to undergo surgery for spinal degenerative diseases. The patients were not under the pressures associated with emergency surgery. However, although the patients are experiencing pain, the long waiting time for surgery may increase the psychological pressure they experience, which may predispose them to POD. At the same time, patients that developed POD were often confirmed to have more pre‐existing diseases, and more preoperative‐related examinations are associated with longer hospital stays.

It is believed that oxidative stress and neuroinflammation play a role in the pathophysiology of POD.^25^, ^39^ According to this study, high preoperative CRP is a risk factor for POD; however, the preoperative ESR variable was excluded when we screened for important features in this study. However, as both CRP and ESR express the degree of inflammation, the ESR may only act as an indirect marker influenced by many factors such as the size, number, and shape of red blood cells. Conversely, CRP is a comprehensive marker of inflammation. It is a stronger inflammatory marker than ESR. According to the LASSO regression, the pulse pressure difference between the normal blood pressure and intraoperative minimum blood pressure is also a risk factor (in this study, the variable of normal blood pressure was replaced by the blood pressure measured on admission). This may be due to either intraoperative hypotension or an excessive drop in blood pressure, acute alterations in cerebral perfusion and oxygenation may expose the brain to the subsequent risk of developing delirium.^40^ However, according to the results of the sensitivity analysis, the effect of PD on the prediction model is negligible, and even Smoking, CCD, and Alcohol had lower Sensitivity Index. Similarly, the results of the SHAP analysis of the XGBoost model show that these four features have very low weights. Considering that previous studies have identified those features may be potential risk factors for POD,^1^, ^41^, ^42^ combined with the conclusions of lasso regression, we finally included those features in the established model.

This study used a machine learning algorithm to build a predictive model to predict the risk of POD in patients with degenerative spinal disorders, which could suggest appropriate preventive measures for patients at risk, particularly by intervening early and correcting abnormal levels of controllable risk factors, and following the multicomponent intervention guidelines to prevent delirium released by The Hospital Elder Life Program (HELP).^43^, ^44^ This has implications for the patient's physical and mental health after surgery, early recovery, and savings in healthcare costs and resources. Using machine learning technology to establish disease prediction and risk‐assessment models can help clinicians better identify the factors that truly affect the prevalence and pathophysiology of diseases.^45^ As future work, we have planned to develop an automated machine learning‐based clinical scoring system based on our dataset and embed it into the clinical case system to provide clinicians with a more practical and easy‐to‐understand tool.

In this study, we developed a POD prediction model with high discrimination. Comparatively with other studies, its prospective validation was another advantage. However, our study had several limitations. First, delirium was divided into manic and silent types. Since this study was a retrospective study, the silent type of delirium is rarely detected or recorded, and the model was only established for those patients exhibiting delirium‐manic manifestations. Second, the study sample was relatively small, and the predictive model requires a larger sample for verification. Third, all the data in this study were derived from the First Affiliated Hospital of Nanchang University. Because of this, other medical institutions may not achieve the same outcomes when using this model. Most probably, when used by another institution, the model may need to be recalibrated, which may alter the exact weights of the features. Finally, this model requires an independent dataset to test the extrapolation and generalization of the model. In the future, we will collect sufficient external validation datasets to further improve this model.

## 5 CONCLUSIONS

In this study, we developed nine different POD prediction models and calibrated them using the AUROC, Brier score, and DCA to select the best‐performing model. The best machine learning algorithm, which was practical and had a good performance, was chosen. This model could achieve an individualized prediction of POD and minimize the cost and risk of delirium preventive measures. We recommend using this model to predict POD and instruct high‐risk patients to take appropriate preventive measures. We believe this model is an important tool for screening patients at high risk of POD among those with degenerative spinal diseases.

## AUTHOR CONTRIBUTIONS

Yu Zhang and Guo‐Mei Zhang conceived and designed the study. Yu Zhang conducted the data collection and wrote the manuscript. Dong‐Hua Wan, Min‐Chen, Yun‐Li Li, Hui Ying, Ge‐Liang Yao, and Zhi‐Li Liu performed the analysis and generated the figures and tables. Guo‐Mei Zhang critically reviewed the manuscript. All authors have read and approved the manuscript.

## FUNDING INFORMATION

This work is supported by the Department of Science and Technology Program of Jiangxi Province, China (No.20212BAG70010).

## CONFLICTS OF INTEREST

The authors declare that they have no competing interest.

## Data Availability

The data that support the findings of this study are available from the corresponding author upon reasonable request.
